# Exploring Host Resistance against Chilli Leaf Curl Disease in a Tolerant Chilli Genotype

**DOI:** 10.3390/plants13121647

**Published:** 2024-06-14

**Authors:** Manisha Mangal, Arpita Srivastava, Bikash Mandal, Vikas Solanki, Shriram J. Mirajkar, Pathour R. Shashank, Pritam Kalia, Jai Chand Rana, Vinod Kumar Sharma

**Affiliations:** 1 Division of Vegetable Science, ICAR—Indian Agricultural Research Institute, New Delhi 110012, Indiashriram.mirajkar@gmail.com (S.J.M.);; 2 Division of Plant Pathology, ICAR—Indian Agricultural Research Institute, New Delhi 110012, Indiavikassinghsolanki@gmail.com (V.S.); 3 Division of Entomology, ICAR—Indian Agricultural Research Institute, New Delhi 110012, India; 4 The Alliance of Bioversity International and CIAT, India Office, New Delhi 110012, India; 5 National Bureau of Plant Genetic Resources, New Delhi 110012, India

**Keywords:** *Begomovirus*, *Bemisia tabaci*, *Capsicum annuum*, vector, virus

## Abstract

In tropical countries, combating leaf curl disease in hot peppers has become important in improvement programs. Leaf curl disease is caused by whitefly (*Bemisia tabaci*) transmitted begomoviruses, which mainly include chilli leaf curl virus (ChiLCV). However, multiple begomoviruses have also been found to be associated with this disease. The *Capsicum annuum* line, DLS-Sel-10, was found to be a tolerant source against this disease during field screening. In this study, we characterized the resistance of DLS-sel-10 against chilli leaf curl virus (ChiLCV) in comparison to the susceptible cultivar Phule Mukta (PM), focusing on the level, stage, and nature of resistance. Comprehensive investigations involved screening of DLS-Sel-10 against the whitefly vector ChiLCV. The putative tolerant line displayed reduced virus infection at the seedling stage, with increasing resistance during vegetative, flowering, and fruiting stages. Both DLS-Sel-10 and PM could be infected with ChiLCV, although DLS-Sel-10 remained symptomless.  Insect feeding assays revealed DLS-Sel-10 as a less preferred host for whiteflies compared to PM. In conclusion, DLS-Sel-10 demonstrated tolerance not only to ChiLCV but also served as an unfavorable host for the whitefly vector. The study highlighted an age-dependent increase in tolerance within DLS-Sel-10, showcasing its potential for effective leaf curl disease management in chilli.

## 1. Introduction

Leaf curl disease is a significant threat to key vegetable crops in tropical and subtropical regions worldwide. This disease is primarily caused by *Begomovirus*, a genus within the *Geminiviridae* family [1,2]. These viruses are transmitted by whiteflies and afflict dicotyledonous plants such as cotton, papaya, tomato, okra, chilli, capsicum, and tobacco, resulting in substantial economic losses for farmers. The genome of *Begomovirus* features circular ssDNA and comprises either two components (DNA-A and DNA-B) or a single monopartite component homologous to the DNA-A component of bipartite viruses [3,4]. The monopartite genome is associated with satellite DNA molecules (beta and alpha satellite) [5].

Chilli, an important vegetable and spice crop in India, is severely affected by various begomoviruses, causing significant economic damage [6,7]. The severity of leaf curl disease has led farmers to abandon the *Kharif* (the main growing season from July to September) cultivation of chilli. Over the past decade, reports of high disease incidence have surfaced, with instances of up to 100% devastation in specific regions [8,9,10]. Symptoms include severe leaf curling, stunted growth, leaf thickening, vein clearing, and reduced fruiting. Chilli cultivation in India during the *Kharif* season coincides with the southwest monsoon. Disease onset typically occurs in late June, about 45–55 days after sowing, escalating rapidly in July, slowing in August, and nearly ceasing by mid-October. Initial signs include vein thickening, followed by leaf curling and thickening. Symptomatic appearance occurs 10–18 days post-infection.

Leaf curl disease in India is known to be a complex disease caused by multiple begomoviruses, including chilli leaf curl virus (ChiLCV), chilli leaf curl India virus (ChiLCINV), chilli leaf curl Vellanad virus (ChiLCVV), tomato leaf curl Joydebpur virus (ToLCJV), tomato leaf curl Bangalore virus (ToLCBaV), tomato leaf curl Palampur virus (ToLCPalV) and tomato leaf curl New Delhi virus (ToLCNDV) [6,8,11,12,13]. Recent studies revealed the association of five distinct *Begomovirus* species (ChiLCV, pepper leaf curl Bangladesh virus (PepLCBV), tomato leaf curl virus (ToLCV), ToLCNDV, papaya leaf curl virus (PaLCuV)) with six different groups of beta satellites (like ToLCBDB, ToLRnB, ChiLCB, ToLCJoB, RaLC, CroYVMB) in diseased plants [6,7]. Most of the leaf curl disease-associated begomoviruses and beta satellites in chilli have been reported to have undergone recombination, which has led to the breakdown of resistance to one or two viruses in otherwise resistant genotypes [14].

Current management involves insecticide spraying for whitefly control, but concerns about pesticide residues have led to a search for sustainable alternatives. Breeding for resistant cultivars is a key solution to address concerns related to leaf curl disease. Identifying resistant/tolerant lines is crucial for resistance breeding programs. Although breeding against leaf curl disease in tomatoes is more advanced than in chillies, some preliminary work, including comparative studies between resistance genes in chilli and tomato [15], study of genetics of resistance to chilli leaf curl disease [16] and identification of wild sources of resistance [17] have been conducted.

To breed for *Begomovirus* resistance, evaluating genetic stocks under natural conditions in the fields with ample virus inoculum and vector populations is necessary. Using this strategy, we have identified three genotypes viz. DLS-Sel-10, WBC-Sel-5, and PBC-142 as putative tolerant lines [18].

Considering the rapid mutational changes in the viral genome, disease control measures initially effective may become ineffective due to viral adaptation [19,20,21,22,23]. Resistance expressed through reduced virus acquisition by the vector and reduced plant inoculation does not exert selection pressure for viral evolution towards higher multiplication rates [24], therefore understanding the nature and dynamics of resistance remains crucial. It is imperative to investigate whether resistance in identified sources is against the vector or the virus and if it is specific to certain plant age stages. This study focused on exploring the resistance in the DLS-Sel-10 line, previously identified as tolerant to leaf curl disease [18]. The investigation aims to determine (i)whether DLS-Sel-10 exhibits tolerance throughout all plant growth stages, (ii) whether the resistance is directed against the vector or the virus, and (iii) whether it confers resistance against the predominant virus, specifically chilli leaf curl virus, responsible for leaf curl in Northern India.

## 2. Results

### 2.1. Effect of Plant Age on Leaf Curl Virus Resistance in DLS-Sel-10

**Figure 1:**
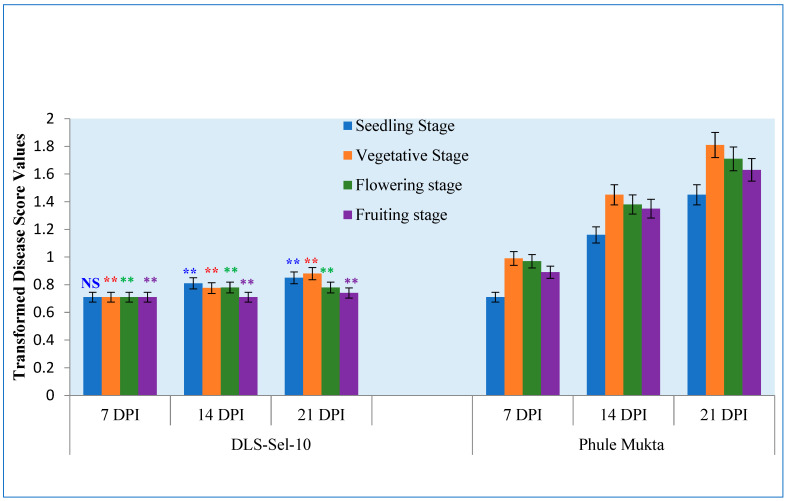
Disease score values of tolerant and susceptible genotypes at different stages under natural epiphytotic conditions. The notation above the respective color bars indicates the level of significance in the data obtained at each stage between DLS-Sel-10 and Phule Mukta. NS: Nonsignificant, **: significant at *p*-value < 0.01.

The comparison of two genotypes for disease score, PM showed a significantly higher disease score as compared to the tolerant genotype at seedling (*p* values 0.5, 0.000027, and <0.00001),vegetative (*p* values 0.001329, <0.00001 and <0.00001), flowering (*p* values 0.001155, <0.00001 and <0.00001) and fruiting stages (*p* values 0.00245, <0.00001, <0.00001) at 7, 14 and 21 DPI respectively (Figure 1). 

### 2.2. Screening for Resistance against Vector

#### 2.2.1. Free Choice Method

The data recorded during the free choice test and presented in Table 1 showed that the development of whitefly was on the abaxial leaf surface. The values of all the parameters, such as number of eggs (34.25 in PM vs. 8.75 in DLS-Sel-10), nymphal count (14.5 in PM vs. 4.25 in DLS-Sel-10), average number of adults (6.25 in PM vs. 0.75 in DLS-Sel-10) as well as the sooty mold growth were found to be significantly higher in PM in comparison to DLS-Sel-10 (Table 1). 

A field experiment was undertaken to assess the white fly population density on the two genotypes at seedling, vegetative, flowering and fruiting stages respectively (Table S1). PM showed higher number of nymphal counts i.e., 24.6, 39.6, 43.8 and 35.0 at seedling, vegetative, flowering and fruiting stage, respectively indicating preference of the vector for this genotype. The nymphal count was statistically at par at different stages of the same genotypes. 

#### 2.2.2. No Choice Method

**Figure 2:**
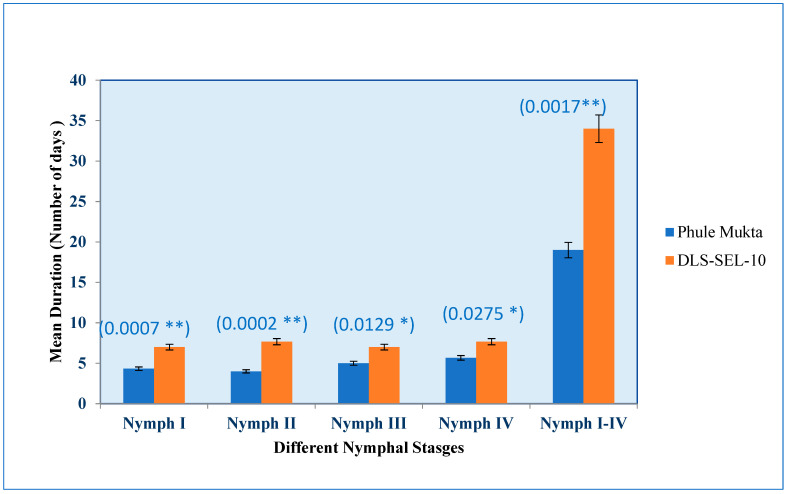
Mean Duration for development of *Bemisia tabaci* throughout the instars on two test lines of chilli. (The values in the parenthesis indicate the *p* values at respective Nymphal stages; **: significant at 1%; * significant at 5%).

The performance of the tolerant genotype DLS-Sel-10 and susceptible PM was evaluated on the basis of the mean duration of development of the nymphs of whiteflies (*Bemisia tabaci*). The mean duration taken by the nymph Ist, IInd, IIIrd, and IVth stages of whiteflies in PM was found to be between 4 to 5.67 days, whereas in DLS-Sel-10 these stages were achieved in 7 to 7.67 days (Figure 2). The growth of whitefly throughout the instars on the test lines was monitored, and found that the total time taken for stage I to pass on to stage II, III, and finally IV was longer in DLS-Sel-10 (34 days against 19 days).

**Figure 3:**
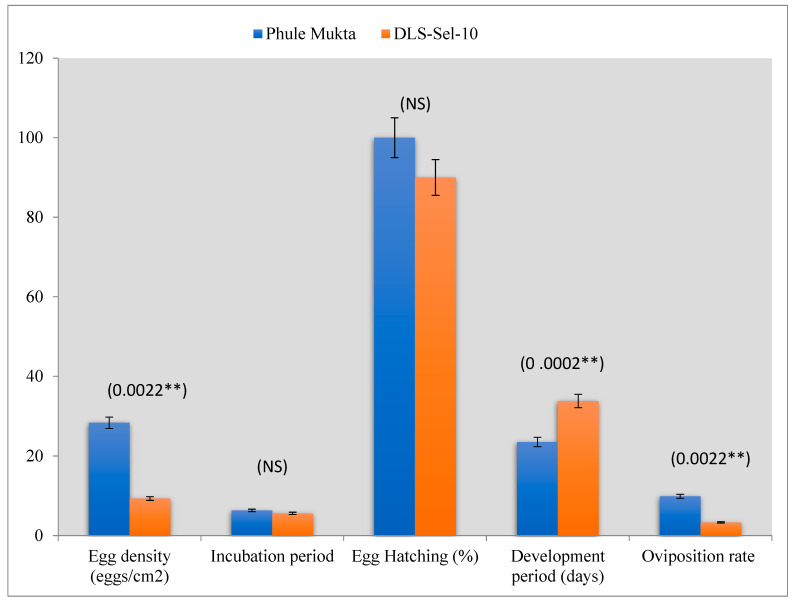
Effect of chilli genotypes on biological parameters of the eggs laid by whiteflies. (The values in the parenthesis indicate the *p* values at respective data points. NS: non significant; **: significant at 1%).

The data on the effects of chilli genotypes on biological parameters of the eggs laid by whiteflies showed that the egg density and incubation period were respectively higher and longer in PM (28.34/cm^2^ and 6.33 days) than in DLS-Sel-10 (9.55/cm^2^) (Figure 3). There was 100% egg hatching on PM as against 90% on DLS-Sel-10. The time required for the development of whitefly was observed to be 23.54 days on PM, whereas it was 33.8 days on DLS-Sel-10. The oviposition rate was also less on DLS-Sel-10 (3.33) than on PM (9.89).

### 2.3. Screening for Resistance Using Challenge Inoculation against ChiLCV

**Figure 4:**
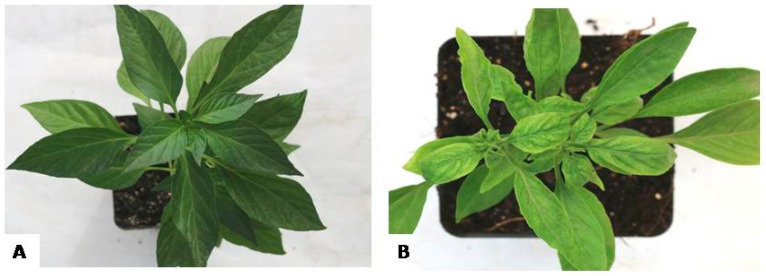
Response of Tolerant/susceptible chilli genotypes after 14 days of infection with ChiLCV ((**A**) No symptom in DLS-Sel-10; (**B**) Leaf curling in Phule Mukta).

There was no symptom development after infection with ChiLCV in DLS-Sel-10 until 35 days of challenge inoculation, while symptoms appeared in PM within 10–12 days (Figure 4). The disease symptom severity increased with time in PM (Table 2).

### 2.4. Detection of Presence of Virus and Viral Titer Load Estimation

**Figure 5:**
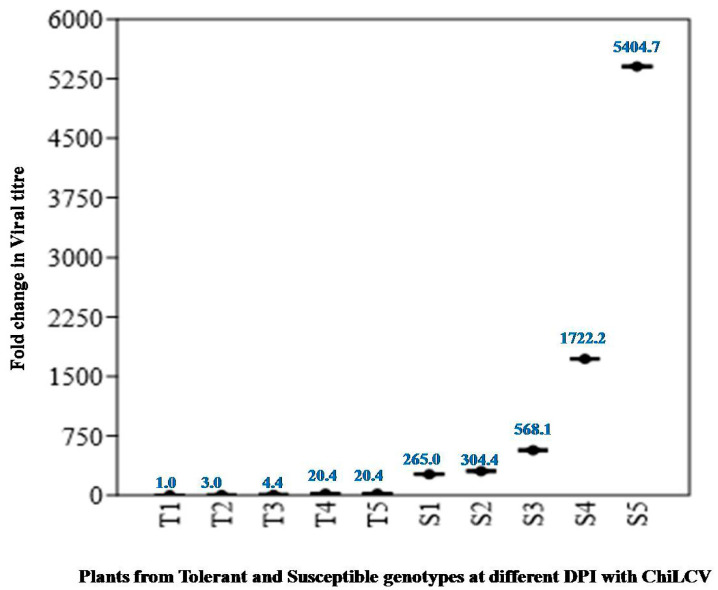
Relative Fold Change in viral titer intolerant and susceptible genotypes at 7, 14, 21, 28, and 35 DPI, respectively, with ChiLCV.

The samples of tolerant and susceptible genotype were tested for the presence of chilli leaf curl virus using ChiLCV specific primers at 7, 14, 21, 28 and 35 DPI. Both DLS-Sel-10 and PM showed presence of virus. Upon qPCR it was found that the viral titer in PM plants after 35 DPI was high (more than 5000 times) as compared to DLS-Sel-10 (Figure 5). 

## 3. Discussion

Chilli leaf curl disease significantly impacts chilli pepper cultivation in India, with various begomoviruses associated with its occurrence [25]. Virus resistance breeding faces challenges due to the rapid mutation of viruses, leading to the breakdown of resistance. An effective strategy involves identifying genes against different virus isolates and pyramiding them for durable horizontal resistance. Identification of pre-dominant viruses involved in leaf curl disease complex and identifying genes against each of these viruses can be useful approaches for breeding against viruses. Our field study revealed ChiLCV as the most dominant virus consistent with other findings [2,6,8,11]. Challenge inoculation experiments demonstrated that ChiLCV could be transmitted to both the genotypes under study. Whitefly nymph counts on genotypes at various growth stages indicated a preference for the susceptible host PM. This preference aligns with studies in the tomato by Fekri et al. [26] who had compared the preference of *Bemisia tabaci* for eight different tomato genotypes and found that the variety CAL-JN3 was the least and Ergon was the most preferred variety by the whitefly. In the present study, the putative tolerant DLS-Sel-10 exhibited approximately two times lower average disease score during the seedling stage compared to the susceptible genotypes PM after 21 DPI. This trend persisted with plant age, reaching its minimum at the flowering and fruiting stages, indicating an age-dependent increase in resistance in the tolerant genotype. Similar responses have been observed in earlier studies on TYLCV-resistant tomato hosts, where semi-dominant genes like *Ty-1* conferred resistance by limiting symptom expression and restricting virus accumulation [27,28,29].

The susceptible genotype PM displayed a significantly higher disease incidence at all stages of plant growth and time points (i.e., 7, 14, and 21 DPI), suggesting susceptibility regardless of genotype age. Nevertheless, a unanimous agreement emerged regarding the observation that both DLS-Sel-10 and PM exhibited the highest disease scores during the vegetative stage, in contrast to the flowering and fruiting stages (Figure 1). Previous studies have also reported that early virus infection in cultivars can lead to more drastic responses than infection at later growth stages in cowpea [30,31]. Gilmer et al. [31] demonstrated that inoculating cowpea cultivars with Cowpea yellow mosaic virus (CpMV) seven days after emergence resulted in a 40–60% yield reduction, compared to a 10–15% loss when plants were inoculated at flowering.

In a free-choice test [32], whiteflies showed a higher preference for the PM genotype over DLS-Sel-10. PM supported greater whitefly growth, indicated by higher egg density, shorter nymphal growth period, increased adult numbers, and more sooty mold growth compared to DLS-Sel-10. Sooty mold, a fungal growth on honeydew secretions, correlates with plant damage. Adult count and sooty mold growth in DLS-Sel-10 indicated reduced whitefly visits in this genotype [33,34].

In a no-choice test, only one host is accessible for the whitefly. If it cannot feed on that host, the growth of the whitefly will be hampered, which will ultimately result in its death [35]. Our results indicated a lower preference of whiteflies for DLS-Sel-10 as compared to PM. This was evidenced by longer hatching time, lower egg density, hatching percent, and oviposition rate on DLS-Sel-10. Selection of the host plant may depend on several factors such as leaf architecture and color [36], leaf pubescence and trichome type and density [37,38], cuticle thickness [39] and compounds that play a role in repelling or attracting whiteflies [40]. Leaf characteristics, including color and thickness, contribute to whitefly host selection. Light yellow-green leaves are believed to be preferred by whiteflies [41,42], explaining the preference for PM over DLS-Sel-10 [36,41,43]. DLS-Sel-10’s dark green, leathery leaves contrast with PM’s pale green, thin leaves, potentially influencing whitefly preference [44].

In our study, DLS-Sel-10 emerged as a less preferred host compared to PM, exhibiting longer nymphal growth periods. Our study highlighted that DLS-Sel-10 showed resistance not only to the vector but also to leaf curl-causing begomoviruses in chilli, with no symptom development despite the presence of ChiLCV. The viral load in DLS-Sel-10 was more than five thousand times lower than the susceptible genotype after 35 DPI, indicating its role as a symptomless carrier and potential virus reservoir. We also found that DLS-Sel-10, despite being a non-preferred host for the whitefly, demonstrated viral acquisition without facilitating subsequent viral multiplication. Consequently, it is inappropriate to categorically deem the host as incompatible with the vector. Our observations suggest the existence of specific host factors impeding viral dominance and symptom development. Similar asymptomatic behavior has been observed in various other tolerant solanaceous hosts of begomoviruses. The first commercial tolerant cultivar, TY20 carrying tolerance from *S. peruvianum*, was a symptomless carrier of TYLCV whether infected in the greenhouse or in the field and showed delayed symptoms [45,46,47]. Notably, various wild tomato species, including *Solanum chilense*, *S. hirsutum*, *S. peruvianum*, and *S. pimpinellifolium*, exhibit analogous asymptomatic carrier behavior following viral infection.

## 4. Materials and Methods

### 4.1. Effect of Plant Age on Resistance

The experiment focused on two genotypes: DLS- Sel-10 (tolerant to leaf curl disease) and PM (a commercially susceptible variety). DLS-Sel-10, developed through pure-line selection in Delhi, demonstrated tolerance against the identified viruses, while PM (a released variety from Mahatma Phule Krishi Vidyapeeth (*MPKV*), Rahuri, India) was susceptible. The morphological features of both genotypes are detailed in Table S2.

### 4.2. Layout of the Experiment

The experiment, conducted at ICAR-IARI New Delhi research farm from June to September, aimed to utilize natural whiteflies. Four net houses and one open plot were used to compare the performance of the tolerant genotype DLS-Sel-10 and susceptible genotype PM in response to whitefly infestation at specific plant ages. Forty-day-old seedlings were transplanted with three replications per net house, each containing five seedlings. The layout is illustrated in Supplementary Figure S1. Net houses were exposed (one net house at one stage) to whiteflies at different stages—vegetative, flowering, and fruiting—while the open plot received whiteflies throughout its growth (serving as a positive control). The plants, once exposed, were left open for the remaining period of growth. The experiment revealed the susceptibility of the studied genotypes to viral infection at various growth stages through whitefly infestation.

In each net house exposed to whiteflies at specific plant growth stages, the tolerant and susceptible genotypes were monitored for leaf curl disease symptoms at 7, 14, and 21 days after opening the nets. Screening for chilli leaf curl disease was performed according to a scale developed by Banerjee et al. [48] and modified by Kumar et al. [49] (Table S3).

### 4.3. Screening for Resistance against Vector

Tolerant genotype DLS-Sel-10 and susceptible genotype PM were assessed for whitefly developmental behavior to understand DLS-Sel-10’s resistance. Two tests, no choice and free choice, were employed for whitefly (*Bemisia tabaci* mitotype Asia II 7) [50] resistance evaluation.

#### 4.3.1. Free Choice Method

For the free-choice test, insect cages in an insect-proof net house contained PM and DLS-Sel-10. The net house protected the test material from external insects, heavy rainfall, and high sunlight intensity. The seeds were sown inside small plastic pots of 4 × 4 square inches filled with cocopeat, vermiculite, and compost mixture in a ratio of 3:1:1. Each of the insect cages consisted of five rows with three plants in each row. The setup had three replications, with each cage having rows of alternating DLS-Sel-10 and PM plants. Non-viruliferous healthy whiteflies (raised and maintained in the laboratory on healthy cucumber plants) were released when the plants developed 5–6 leaves. Population development was monitored by counting adult whiteflies, egg masses, and nymphs and assessing parameters like sooty mold growth. During the first week of inoculation, all plants in the cage were shaken twice a day in order to distribute the whiteflies to feed on all the plants available in that cage. Observations were taken at 7-day intervals for a month.

Observations of a number of adult whiteflies were taken by directly counting them on the abaxial surface of the 3rd and 6th leaf from the top (Figure S2). Egg mass and nymphal count were determined by placing the leaves under the stereo microscope (10×).

Scoring of sooty-mold growth was made according to visual appearance on a scale of 0–4 [33,34]. For sooty-mold growth scores were: (0) sooty mold absent, (1) some sooty mold present on one leaf, (2) sooty mold present on two or more leaves, (3) heavy sooty mold (thick and covering 10% of leaflet area) present on one or two leaves (4) heavy sooty mold present on more than three leaves.

Data on adults, eggs, nymphs, and leaf areas in the free-choice test were used to determine.

- Adult whitefly density = number of adult whiteflies/cm^2^ of leaf
- Egg density = number of eggs/cm^2^ of leaf,
- Nymphal density = number of nymphs/cm^2^ of leaf.

#### 4.3.2. Sampling of Whiteflies to Determine Whitefly Population Density

Sampling was done on five plants of resistant genotype and five of susceptible for each set of experiments, including open plot weekly. Plants were chosen at random, taking care not to single out tall or heavily infested plants. Once a plant was selected, leaf samples were taken from the top, middle, and lower portion of the plant during evening hours (between 4.00–6.00 PM, IST); caution was taken to avoid sampling within 24 h after rain, as it may not accurately reflect whitefly densities. The leaf was tallied as “infested” if it contained three or more whitefly adults and as uninfested if it contained less than 3. The collected leaf samples were also observed under a microscope for the number of nymphal counts.

#### 4.3.3. No Choice Method

For the no-choice test conducted in an insect-proof chamber, clip-on cages were used to force whiteflies onto a specific genotype. Seeds of the two tested genotypes were sown in pots with compost, vermiculite, and compost mixture (3:1:1). The temperature in the compartments was maintained at 25°/16 °C (day/night), photoperiod at 16 h light/8 h dark and relative humidity was kept at 70%. After one month of transplanting to mud pots with peat-moss soil, ten plants (5 of each genotype) were challenge-infested with non-viruliferous whitefly adults sedated with CO_2_. Clip-on cages (with a diameter of 2 cm × height of 1 cm) were placed on the abaxial surface of a leaflet, and 3–5 cages were attached per plant. For the experiment, five whiteflies (two male and three female (n)) were released into each clip-on cage (Figure S3).

Two days after infestation, the clip-on cages were removed, and living and dead whiteflies were counted. Daily observations were made to record developmental changes, including nymphal growth stages, which were categorized into four instars based on the illustration for instar identification given by Chaubey et al. [51]. The assessed biological parameters were: nymphal stage (duration, survival, number of instars, and duration of each instar); adult stage (oviposition period, number of eggs per female); egg stage (duration, survival); total cycle (time span from egg to adult emergence and survival). The number of eggs (e) was counted under a stereo microscope (10× magnification). The emerging adults (a_i_) were counted and removed from the cages every day (t_i_) for a week. Pupal cases (p) were counted seven days after the first-emerging adult whiteflies. Oviposition rate (OR) and development periods (DP) were calculated by using the equations [52] shown below.

$$OR=\frac{2e}{d(m+n)}\left(eggs/female/day\right)$$

$$DP=\frac{\sum t_{i}-a_{i}}{\sum a_{i}}$$

where d = number of days = 2; m = number of surviving adults and n = number of adults used for inoculation = 5.

Means of all observations were calculated in each replication and analyzed by method given by Panse and Sukhatme [53]. To assess the statistical significance within the overall variance, analysis of variance (ANOVA) was conducted, subsequently employing the Fisher’s Least Significant Difference (LSD) method to evaluate any significant disparities in the mean values.

### 4.4. Screening for Resistance against Virus

#### 4.4.1. Experimental Setup

To assess the behavior of the DLS-Sel-10 line against ChiLCV, the experiment was conducted using viruliferous whiteflies. The whiteflies were reared on brinjal plants (Figure S4A) under controlled conditions (28–35 °C temperature, 30–50% relative humidity, and 14 h photoperiod). The tolerant genotype DLS-Sel-10 and the susceptible genotype PM were screened by exposing them to whiteflies carrying ChiLCV. The whiteflies were made viruliferous by allowing them to feed on infected chilli plants (having pure isolates of each virus) for 24 h (Figure S4B). Plastic cages were used for screening (Figure S4C), and the plants were observed for symptom development after inoculation with ChiLCV-fed whiteflies. Five viruliferous whiteflies were released on each of the test plants. Additionally, mock samples were included where genotypes were exposed to nonviruliferous whiteflies (five whiteflies/plant) with no virus. After two days of whitefly feeding on healthy seedlings, the whiteflies were killed by spraying spiromeficin at the rate of 0.5 mL/ltr. Screening involved regular observations for symptom development at weekly intervals.

#### 4.4.2. Detection of Presence of Virus and Viral Titer Estimation

After challenging DLS-Sel-10 and PM with viruliferous whiteflies, plant samples were screened for virus presence using virus-specific primers, and q-PCR was conducted to estimate viral titers in both tolerant and susceptible genotypes. Leaf samples from both the genotypes after 7, 14, 21, 28 and 35 days of infection with whiteflies fed on ChiLCV-infected plants (24 h) were collected for DNA extraction (Begomoviruses being ssDNA viruses). Genomic DNA was extracted from young leaf tissue (top four leaves) of each genotype following the C-TAB method [54]. In the experiment, T1, T2, T3, T4, and T5 indicate infected plants of tolerant genotype DLS-Sel-10 after 7, 14, 21, 28 and 35 days of infection while S1, S2, S3, S4, and S5 indicate infected plants of the susceptible PM at respective stages. Viral titers were quantified using a relative quantification approach, wherein plant samples from tolerant genotype after 7 seven days of infection (T1) were used as calibrators or the reference sample for calculating ΔΔCt values. Three biological replicates per disease score were employed, and Ca-actin [55] served as the housekeeping gene. Primers for estimation of viral titer were designed from the AC1/AC4 region of the Chilli Leaf Curl Virus genome. The sequence of the forward primer of ChiLCV was 5′ CGGCATATGCGTCGTTGGC-AGAC 3′ while that of the reverse primer was 5′ TTCTTCGACCTCGTTTCCCCAACC 3′. The sequence of the internal control Actin was FP 5′ GAAGCTCAATCCAAACGTGGTATT 3′ and RP 5′ CTCAAACATGATTTGTGTCATC 3′.

Three technical replicates were run in real-time PCR to account for pipetting errors. For qRT-PCR, 5 µL of SYBR Green master mix (Applied Biosystem, Foster City, CA, USA), 3 μL nuclease-free water, and 1 μL (200 nm) each of forward and reverse primer of the desired gene was used. PCR program comprised of initial denaturation at 94 °C for 5 min, 40 cycles of denaturation at 94 °C for 15 s, annealing at 55 °C for 35 s, and extension at 72 °C for 35 s. The ΔCt of the target gene was normalized with internal control Ca-actin. The ΔCt values were used to plot the graph to obtain the relative titer of the virus in plants with different disease scores.

## 5. Conclusions

This study contributes valuable insights into host resistance/tolerance against leaf curl disease in chilli. The response of the identified tolerant source, DLS-Sel-10, to leaf curl disease as compared to the susceptible host, PM, revealed that ChiLCV was detected in both genotypes during challenge inoculation. However, DLS-Sel-10 was identified as a symptomless carrier of ChiLCV, showing not only resistance to ChiLCV multiplication but also resistance against the vector whitefly. DLS-Sel-10 was also a non-preferred host for whiteflies compared to PM during screening.

This study also sheds light on the differential response of various plant growth stages to viral infestation in tolerant and susceptible lines. The resistance in the tolerant line was observed to increase with the plant’s age, while susceptibility in the susceptible genotype appeared to be independent of the genotype’s age. These findings enhance our understanding of the intricate dynamics between plant age, resistance, and susceptibility in the context of leaf curl disease in chilli.

## Figures and Tables

**Table 1:** Biological Parameters of Whitefly in Free Choice Method.

Test Genotypes	Number of Egg	Nymphal Count	Average No. of Adults	Sooty Mold Growth
Phule Mukta	34.25	14.5	6.25	2.5
DLS-Sel-10	8.75	4.25	0.75	0.25
CD	15.994	5.151	2.563	0.975
SEM	4.53	1.46	0.726	0.27

CD indicates Critical Difference, and SEM indicates Standard Error of Mean.

**Table 2:** Response of tolerant and susceptible chilli genotypes to ChiLCV infection.

Days after Inoculation	Disease Score	
	Infection with ChiLCV	
	Phule Mukta	DLS-Sel-10
7 days	0	0
14 days	2	0
21 days	3	0
28 days	4	0
35 days	4	0

## Data Availability

Data are contained within the article.

## Supplementary Materials

The following supporting information can be downloaded at: https://www.mdpi.com/article/10.3390/plants13121647/s1, Table S1. Average Nymphal count at different developmental stages in tolerant and suscepltible chilli genotypes. Table S2. Plant morphological features of Phule Mukta and DLS-Sel-10. Table S3. Infection type classification given by Banerjee 1987 and modified by Kumar et al., 2006. Figure S1. Lay out of experiment for identifying the most sensitive stage of the plant for leaf curl disease. Figure S2. Whitefly on abaxial leaf surface in Free choice method. Figure S3. Layout of experiment (A) and placement of clip on cages on abaxial surface of DLS-Sel-10 (B). Figure S4. Screening for resistance against virus. (A) Whitefly rearing on brinjal plants; (B) Acquisition of whiteflies on infected plants of hot pepper with pure isolate of one virus; (C) Set up for release of whiteflies on each single plant.
