# Supplementary material for: Exploring Host Resistance against Chilli Leaf Curl Disease in a Tolerant Chilli Genotype
Source: Plants (Basel). 2024 Jun 14;13(12):1647. doi: 10.3390/plants13121647 (PMC11207214; doi:10.3390/plants13121647)
Supplement: Supplementary file 1 [file plants-13-01647-s001.zip › plants-2975055-supplementary.pdf]

Table S1. Average Nymphal count at different developmental stages in tolerant and suscepltible chilli genotypes.

Treatment	Seedling stage		Vegetative stage		Flowering stage		Fruiting stage	
	Mean	S.E.	Mean	S.E.	Mean	S.E.	Mean	S.E.
DLS-SEl-10	13.80	2.08	15.20	1.74	16.60	1.57	19.40	1.44
Phule Mukta	24.60	2.01	39.60	1.47	43.80	3.63	35.00	2.97
C.D.	8.49		7.33		9.12		8.242	
SEM	2.57		2.21		2.75		2.489	
SD	3.63		3.13		3.89		3.519	
C.V.	31.51		18.59		21.27		21.403	

* Total nymphal count of three leaves (average of five plants). CD: Critical Difference; S.E.: Standard Error; SEM: Standard Error of Mean; SD: Standard Deviation; C.V.: Coefficient of Variation.

Table S2. Plant morphological features of Phule Mukta and DLS-Sel-10.

Plant Characteristics	Phule Mukta	DLS-Sel-10
Plant Height	53–57 cm	65–70 cm
Fruiting Habit	Single pendant	Cluster erect
Fruit length	6–7 cm	5–6 cm
Leaf colour	Pale green	Dark green
Plant Habit	Semi-erect	Erect
Canopy density	Open type	Dense canopy

Table S3. Infection type classification given by Banerjee 1987 and modified by Kumar *et al.*, 2006.

Class	Grade	Description of symptoms
Immune	0	No symptom
Highly resistant	1	0 to 5% curling and clearing of upper leaves
Resistant	2	6 to 25% curling, clearing of leaves and swelling of veins
Moderately susceptible	3	26 to 50% curling, puckering and yellowing of leaves and swelling of veins
Susceptible	4	51 to 75% leaf curling and stunted plant growth and blistering of internodes
Highly susceptible	5	More than 75% curling and deformed small leaves, stunted plant growth with small flowers and no or small fruit set

Net house 1		Net house 2		Net house 3		Net house 4		Open Plot		
x	o	x	o	x	o	x	o	x	o	Replication 1
x	o	x	o	x	o	x	o	x	o	
x	o	x	o	x	o	x	o	x	o	
x	o	x	o	x	o	x	o	x	o	
x	o	x	o	x	o	x	o	x	o	
o	x	o	x	o	x	o	x	o	x	Replication 2
o	x	o	x	o	x	o	x	o	x	
o	x	o	x	o	x	o	x	o	x	
o	x	o	x	o	x	o	x	o	x	
o	x	o	x	o	x	o	x	o	x	
x	o	x	o	x	o	x	o	x	o	Replication 3
x	o	x	o	x	o	x	o	x	o	
x	o	x	o	x	o	x	o	x	o	
x	o	x	o	x	o	x	o	x	o	
x	o	x	o	x	o	x	o	x	o	
Never exposed to white fly infestation (Negative Control)		Exposed to natural infestation by whitefly at vegetative branching stage		Exposed to natural white fly infestation at flower initiation stage		Exposed to natural whitefly infestation at fruiting stage		Exposed to natural whitefly infestation throughout their life span (positive Control)		

Total plants/net house= 30 (3 replications of each genotype with 5 plants per replication); X= Tolerant line DLS-Sel-10; O= Susceptible line Phule Mukta

Figure S1. Lay out of experiment for identifying the most sensitive stage of the plant for leaf curl disease.

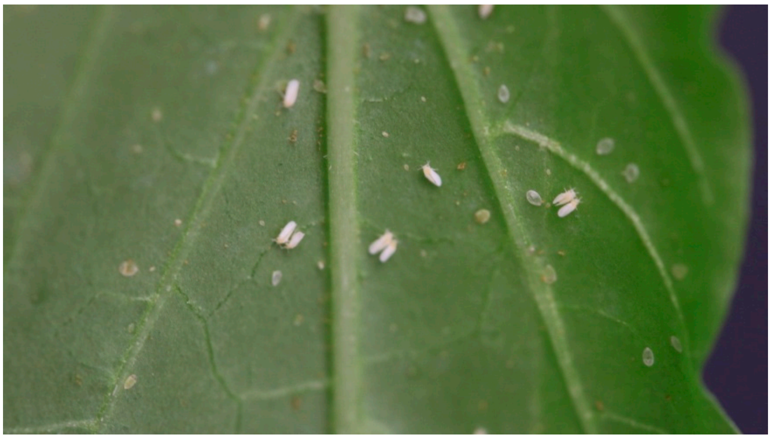

Figure S2. Whitefly on abaxial leaf surface in Free choice method.

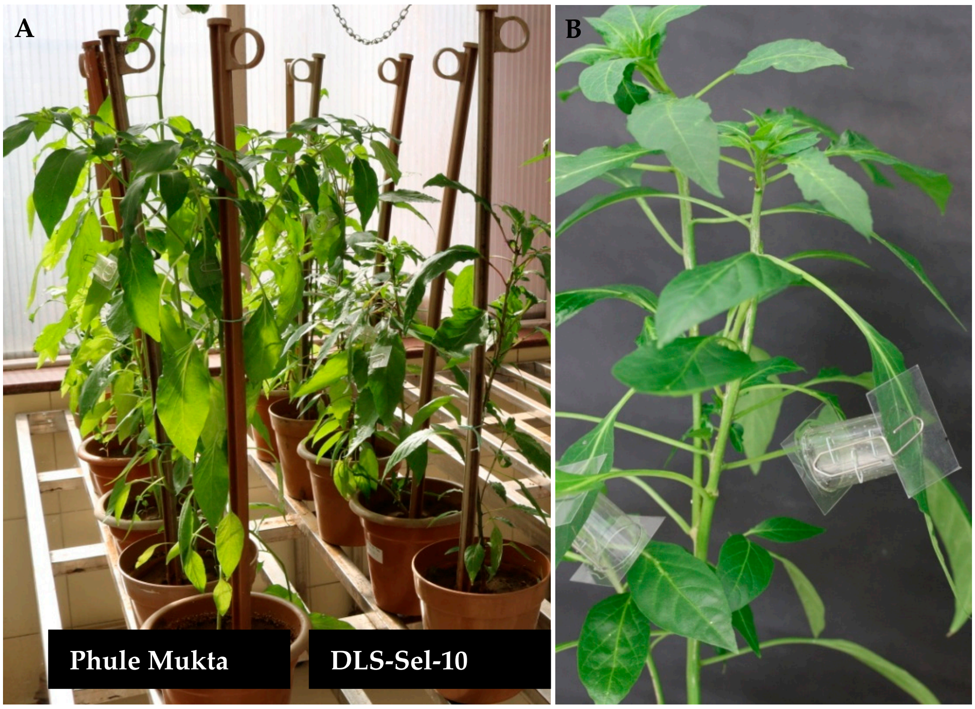

Figure S3. Layout of experiment (**A**) and placement of clip on cages on abaxial surface of DLS-Sel-10 (**B**).

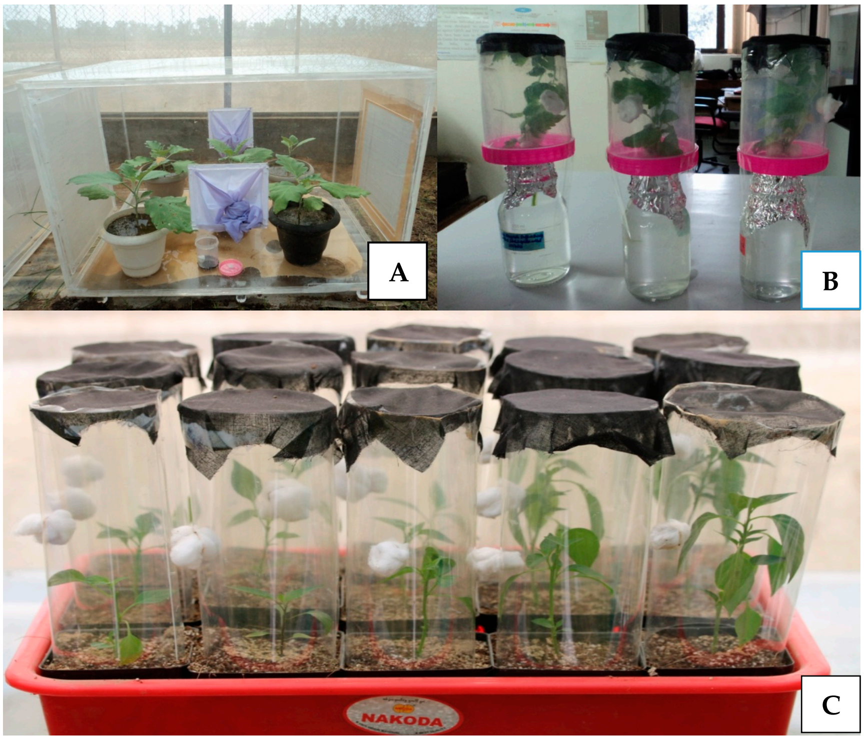

Figure S4. Screening for resistance against virus. (**A**) Whitefly rearing on brinjal plants; (**B**) Acquisition of whiteflies on infected plants of hot pepper with pure isolate of one virus; (**C**) Set up for release of whiteflies on each single plant.
